# Supplementary material for: Obesity in the Balinese is associated with FTO rs9939609 and rs1421085 single nucleotide polymorphisms
Source: PeerJ. 2020 Jan 3;8:e8327. doi: 10.7717/peerj.8327 (PMC6944099; doi:10.7717/peerj.8327)
Supplement: Supplemental Information 1 [file peerj-08-8327-s001.docx]

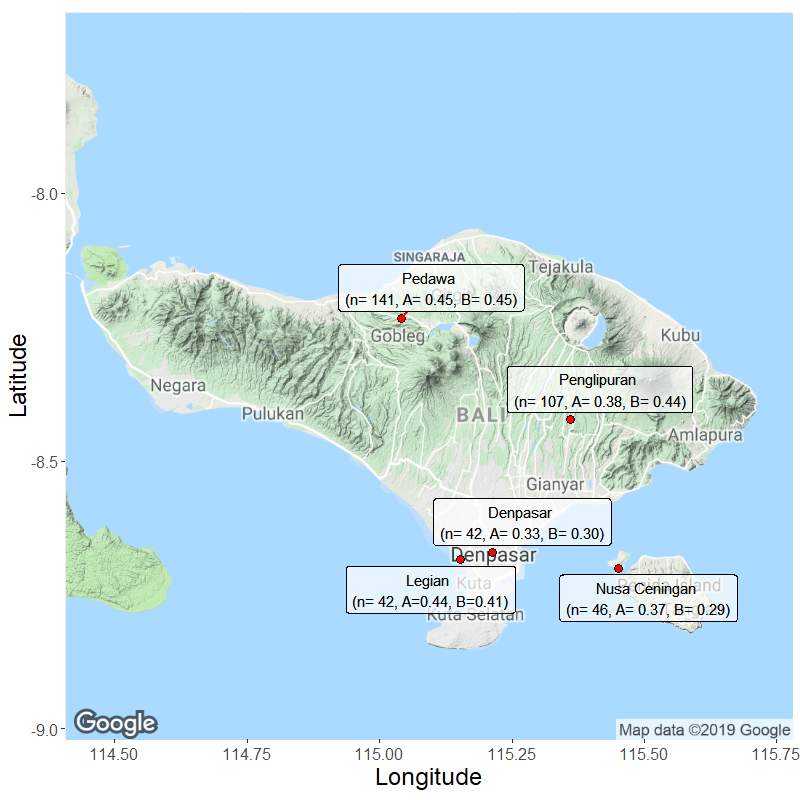


Figure S1. Map and sample size of the five locations. n: sample size; A= MAF of rs9939609, B= MAF of rs1421085. Map data © 2019 Google.

Table S1. Outer and inner primers for ARMS-PCR and ARMS-PCR results

| **SNP** | **Primers** | **Fragments** |
| --- | --- | --- |
| rs9939609 | Fin: 5’- TAGGTTCCTTGCGACTGCTGTGAATATA-3’  Rin: 5’-GAGTAACAGAGACTATCCAAGTGCATCTCA-3’  Fout: 5’- GTTCTACAGTTCCAGTCATTTTTGACAGC-3’  Rout: 5’- AGCCTCTCTACCATCTTATGTCCAAACA-3’ | TT: 436 bp, 293 bp  TA: 436 bp, 293 bp, 201 bp  AA: 436 bp, 201 bp |
| rs1421085 | Fin: 5’- TAGCAGTTCAGGTCCTAAGGCATTAT-3’  Rin: 5’- ACAAATTCTCATCAGACACTTAATCACTG-3’  Fout: 5’- TTTAGGTTGTAATGAAGTTTTAGGCCTC-3’  Rout: 5’- ATCAGGTTAAATAAATGCTTCTGGACAG-3’ | TT: 446 bp, 205 bp  TC: 446 bp, 295 bp, 205 bp  CC: 446 bp, 295 bp |

Table S2. Models of FTO SNPs associations with obesity parameters

| **N = 612** | **BMI** | | **WC** | | **WHtR** | |
| --- | --- | --- | --- | --- | --- | --- |
|  | *Estimates* | *p* | *Estimates* | *p* | *Estimates* | *p* |
| **FTO rs9939609** |  |  |  |  |  |  |
| **Codominant Model** |  |  |  |  |  |  |
| TA | 0.29 | 0.492 | 1.45 | 0.132 | 0.01 | 0.162 |
| AA | 1.42 | **0.010** | 2.36 | 0.066 | 0.01 | 0.152 |
| Age (years) | -0.01 | 0.467 | 0.01 | 0.673 | <0.01 | **0.013** |
| Male | 0.81 | 0.031 | 5.22 | **<0.001** | <0.01 | 0.440 |
| Urban | 2.75 | **<0.001** | 7.59 | **<0.001** | 0.04 | **<0.001** |
| **Dominant Model** |  | |  | |  | |
| TA+AA | 0.58 | 0.140 | 1.69 | 0.064 | 0.01 | 0.105 |
| Age (years) | -0.01 | 0.476 | 0.01 | 0.670 | <0.01 | **0.013** |
| Male | 0.85 | **0.024** | 5.25 | **<0.001** | <0.01 | 0.451 |
| Urban | 2.84 | **<0.001** | 7.66 | **<0.001** | 0.04 | **<0.001** |
| **Recessive Model** |  | |  | |  | |
| AA | 1.25 | **0.012** | 1.51 | 0.190 | 0.01 | 0.363 |
| Age (years) | -0.01 | 0.482 | 0.01 | 0.633 | <0.01 | **0.011** |
| Male | 0.82 | 0.029 | 5.26 | **<0.001** | <0.01 | 0.467 |
| Urban | 2.73 | **<0.001** | 7.49 | **<0.001** | 0.03 | **<0.001** |
| **FTO rs1421085** |  |  |  |  |  |  |
| **Codominant Model** |  |  |  |  |  |  |
| TC | 0.54 | 0.190 | 2.01 | 0.035 | 0.01 | 0.042 |
| CC | 1.41 | **0.009** | 2.97 | **0.017** | 0.02 | 0.032 |
| Age (years) | -0.01 | 0.452 | 0.01 | 0.65 | <0.01 | **0.012** |
| Male | 0.81 | 0.030 | 5.21 | **<0.001** | <0.01 | 0.425 |
| Urban | 2.84 | **<0.001** | 7.70 | **<0.001** | 0.04 | **<0.001** |
| **Dominant Model** |  | |  | |  | |
| TC+CC | 0.78 | 0.043 | 2.28 | **0.011** | 0.01 | **0.016** |
| Age (years) | -0.01 | 0.511 | 0.02 | 0.618 | <0.01 | **0.010** |
| Male | 0.85 | **0.024** | 5.25 | **<0.001** | <0.01 | 0.445 |
| Urban | 2.87 | **<0.001** | 7.73 | **<0.001** | 0.04 | **<0.001** |
| **Recessive Model** |  | |  | |  | |
| CC | 1.12 | **0.022** | 1.86 | 0.102 | 0.01 | 0.156 |
| Age (years) | -0.01 | 0.438 | 0.01 | 0.679 | <0.01 | **0.013** |
| Male | 0.82 | 0.029 | 5.24 | **<0.001** | <0.01 | 0.444 |
| Urban | 2.80 | **<0.001** | 7.56 | **<0.001** | 0.03 | **<0.001** |

Statistical analysis was done using linear regression model, with adjustments for age, sex (male/female) and population (urban/rural). BMI: body mass index, WC: waist circumference, WHtR: waist to height ratio. The significant *p* values after Bonferroni correction are indicated in bold (*p* < 0.025).

Table S3. Models of FTO SNPs associations with obesity categories

| **N = 612** | **High BMI** | | **High WC** | | **High WHtR** | |
| --- | --- | --- | --- | --- | --- | --- |
|  | *Odds Ratio* | *p* | *Odds Ratio* | *p* | *Odds Ratio* | *p* |
| **FTO rs9939609** |  |  |  |  |  |  |
| **Codominant Model** |  |  |  |  |  |  |
| TA | 0.96 | 0.827 | 1.12 | 0.561 | 1.35 | 0.115 |
| AA | 1.55 | 0.086 | 1.40 | 0.181 | 1.40 | 0.201 |
| Age (years) | 1.00 | 0.808 | 1.01 | 0.266 | 1.00 | 0.582 |
| Male | 1.67 | **0.004** | 0.53 | **<0.001** | 0.93 | 0.676 |
| Urban | 3.13 | **<0.001** | 2.79 | **<0.001** | 3.01 | **<0.001** |
| **Dominant Model** |  | |  | |  | |
| TA+AA | 1.09 | 0.628 | 1.18 | 0.345 | 1.37 | 0.087 |
| Age (years) | 1.00 | 0.802 | 1.01 | 0.263 | 1.00 | 0.581 |
| Male | 1.70 | **0.003** | 0.54 | <0.001 | 0.93 | 0.680 |
| Urban | 3.23 | **<0.001** | 2.84 | <0.001 | 3.02 | **<0.001** |
| **Recessive Model** |  | |  | |  | |
| AA | 1.59 | 0.042 | 1.31 | 0.228 | 1.17 | 0.506 |
| Age (years) | 1.00 | 0.800 | 1.01 | 0.260 | 1.00 | 0.546 |
| Male | 1.67 | **0.004** | 0.53 | **<0.001** | 0.94 | 0.714 |
| Urban | 3.14 | **<0.001** | 2.77 | **<0.001** | 2.93 | **<0.001** |
| **FTO rs1421085** |  |  |  |  |  |  |
| **Codominant Model** |  |  |  |  |  |  |
| TC | 1.09 | 0.657 | 1.21 | 0.316 | 1.47 | 0.045 |
| CC | 1.65 | 0.048 | 1.69 | 0.034 | 1.84 | **0.018** |
| Age (years) | 1.00 | 0.750 | 1.01 | 0.283 | 1.00 | 0.569 |
| Male | 1.67 | **0.005** | 0.53 | **<0.001** | 0.92 | 0.638 |
| Urban | 3.24 | **<0.001** | 2.86 | **<0.001** | 3.07 | **<0.001** |
| **Dominant Model** |  | |  | |  | |
| TC+CC | 1.23 | 0.263 | 1.33 | 0.109 | 1.56 | **0.013** |
| Age (years) | 1.00 | 0.815 | 1.01 | 0.247 | 1.00 | 0.533 |
| Male | 1.70 | **0.003** | 0.53 | **<0.001** | 0.93 | 0.671 |
| Urban | 3.26 | **<0.001** | 2.88 | **<0.001** | 3.08 | **<0.001** |
| **Recessive Model** |  | |  | |  | |
| CC | 1.57 | 0.047 | 1.52 | 0.060 | 1.49 | 0.090 |
| Age (years) | 1.00 | 0.745 | 1.01 | 0.294 | 1.00 | 0.600 |
| Male | 1.67 | **0.004** | 0.53 | **<0.001** | 0.93 | 0.664 |
| Urban | 3.22 | **<0.001** | 2.81 | **<0.001** | 2.96 | **<0.001** |

Statistical analysis was done using logistic regression model, with adjustments for age, sex (male/female) and population (urban/rural). BMI: body mass index, WC: waist circumference, WHtR: waist to height ratio. High BMI: BMI ≥ 25, High WC: male’s wc ≥ 90 cm or female’s wc ≥ 80 cm, High WHtR: WHtR ≥ 0.5. The significant *p* values after Bonferroni correction are indicated in bold (*p* < 0.025).

Table S4. Models of FTO SNPs associations with obesity parameters in male vs. female

|  | **Male (n = 326)** | | | | | | **Female (n = 286)** | | | | | |
| --- | --- | --- | --- | --- | --- | --- | --- | --- | --- | --- | --- | --- |
|  | **BMI** | | **WC** | | **WHtR** | | **BMI** | | **WC** | | **WHtR** | |
|  | *Estimates* | *p* | *Estimates* | *p* | *Estimates* | *p* | *Estimates* | *p* | *Estimates* | *p* | *Estimates* | *p* |
| **FTO rs9939609** | | | | | | | | | | | | |
| **Codominant Model** | | | | | | | | | | | | |
| TA | 0.69 | 0.192 | 0.96 | 0.466 | <0.01 | 0.586 | -0.18 | 0.782 | 1.85 | 0.191 | 0.01 | 0.180 |
| AA | 1.10 | 0.100 | 1.41 | 0.398 | <0.01 | 0.670 | 1.87 | 0.044 | 3.16 | 0.115 | 0.02 | 0.144 |
| Age (years) | -0.02 | 0.184 | -0.04 | 0.34 | <0.01 | 0.332 | -0.01 | 0.729 | 0.04 | 0.415 | <0.01 | 0.064 |
| Urban | 3.49 | **<0.001** | 9.38 | **<0.001** | 0.04 | **<0.001** | 1.90 | **0.004** | 5.66 | **<0.001** | 0.02 | **0.011** |
| **Dominant Model** | | | | | | | | | | | | |
| TA+AA | 0.80 | 0.105 | 1.09 | 0.380 | <0.01 | 0.563 | 0.29 | 0.644 | 2.15 | 0.109 | 0.01 | 0.112 |
| Age (years) | -0.02 | 0.175 | -0.04 | 0.333 | <0.01 | 0.331 | <0.01 | 0.821 | 0.04 | 0.394 | <0.01 | 0.059 |
| Urban | 3.53 | **<0.001** | 9.42 | **<0.001** | 0.04 | **<0.001** | 2.05 | **0.002** | 5.76 | **<0.001** | 0.02 | **0.009** |
| **Recessive Model** | | | | | | | | | | | | |
| AA | 0.69 | 0.242 | 0.83 | 0.569 | <0.01 | 0.848 | 1.97 | **0.021** | 2.11 | 0.251 | 0.01 | 0.314 |
| Age (years) | -0.02 | 0.186 | -0.04 | 0.341 | <0.01 | 0.331 | -0.01 | 0.715 | 0.04 | 0.369 | <0.01 | 0.053 |
| Urban | 3.44 | **<0.001** | 9.32 | **<0.001** | 0.04 | **<0.001** | 1.91 | **0.003** | 5.53 | **<0.001** | 0.02 | **0.014** |
| **FTO rs1421085** | | | | | | | | | | | | |
| **Codominant Model** | | | | | | | | | | | | |
| TC | 0.95 | 0.069 | 1.76 | 0.180 | 0.01 | 0.233 | 0.07 | 0.911 | 2.11 | 0.131 | 0.01 | 0.109 |
| CC | 0.76 | 0.237 | 1.59 | 0.323 | 0.01 | 0.478 | 2.39 | **0.009** | 4.65 | **0.019** | 0.03 | **0.021** |
| Age (years) | -0.02 | 0.232 | -0.04 | 0.395 | <0.01 | 0.283 | -0.01 | 0.689 | 0.03 | 0.449 | <0.01 | 0.073 |
| Urban | 3.57 | **<0.001** | 9.51 | **<0.001** | 0.05 | **<0.001** | 2.04 | **0.002** | 5.74 | **<0.001** | 0.02 | **0.009** |
| **Dominant Model** | | | | | | | | | | | | |
| TC+CC | 0.89 | 0.066 | 1.70 | 0.160 | 0.01 | 0.239 | 0.62 | 0.316 | 2.71 | **0.041** | 0.02 | **0.035** |
| Age (years) | -0.02 | 0.223 | -0.04 | 0.389 | <0.01 | 0.289 | -0.01 | 0.787 | 0.04 | 0.412 | <0.01 | 0.064 |
| Urban | 3.56 | **<0.001** | 9.50 | **<0.001** | 0.05 | **<0.001** | 2.06 | **0.002** | 5.76 | **<0.001** | 0.02 | **0.008** |
| **Recessive Model** | | | | | | | | | | | | |
| CC | 0.23 | 0.688 | 0.61 | 0.670 | <0.01 | 0.850 | 2.35 | **0.005** | 3.48 | 0.056 | 0.02 | 0.067 |
| Age (years) | -0.02 | 0.168 | -0.04 | 0.323 | <0.01 | 0.337 | -0.01 | 0.693 | 0.04 | 0.395 | <0.01 | 0.059 |
| Urban | 3.49 | **<0.001** | 9.36 | **<0.001** | 0.04 | **<0.001** | 2.03 | **0.002** | 5.66 | **<0.001** | 0.02 | **0.010** |

Statistical analysis was done using linear regression model, with adjustments for age and population (urban/rural). BMI: body mass index, WC: waist circumference, WHtR: waist to height ratio. The significant *p* values after Bonferroni correction are indicated in bold (*p* < 0.025).

Table S5. Models of FTO SNPs associations with obesity categories in male vs. female

|  | **Male (n = 326)** | | | | | | **Female (n = 286)** | | | | | |  |  |  |  |  |  |  |  |  |  |  |  |  |  |  |  |
| --- | --- | --- | --- | --- | --- | --- | --- | --- | --- | --- | --- | --- | --- | --- | --- | --- | --- | --- | --- | --- | --- | --- | --- | --- | --- | --- | --- | --- |
|  | **High BMI** | | **High WC** | | **High WHtR** | | **High BMI** | | **High WC** | | **High WHtR** | |  |  |  |  |  |  |  |  |  |  |  |  |  |  |  |  |
|  | *Odds Ratio* | *p* | *Odds Ratio* | *p* | *Odds Ratio* | *p* | *Odds Ratio* | *p* | *Odds Ratio* | *p* | *Odds Ratio* | *p* |  |  |  |  |  |  |  |  |  |  |  |  |  |  |  |  |
| **FTO rs9939609** | | | | | | | | | | | | |  |  |  |  |  |  |  |  |  |  |  |  |  |  |  |  |
| **Codominant Model** | | | | | | | | | | | | |  |  |  |  |  |  |  |  |  |  |  |  |  |  |  |  |
| TA | 1.14 | 0.643 | 1.13 | 0.661 | 1.28 | 0.363 | 0.76 | 0.347 | 1.09 | 0.757 | 1.41 | 0.205 |  |  |  |  |  |  |  |  |  |  |  |  |  |  |  |  |
| AA | 1.10 | 0.781 | 1.19 | 0.608 | 1.26 | 0.514 | 2.42 | **0.022** | 1.69 | 0.180 | 1.48 | 0.321 |  |  |  |  |  |  |  |  |  |  |  |  |  |  |  |  |
| Age (years) | 0.99 | 0.363 | 1.00 | 0.996 | 0.99 | 0.376 | 1.00 | 0.935 | 1.01 | 0.268 | 1.01 | 0.227 |  |  |  |  |  |  |  |  |  |  |  |  |  |  |  |  |
| Urban | 4.47 | **<0.001** | 3.44 | **<0.001** | 3.69 | **<0.001** | 2.00 | **0.019** | 2.27 | **0.002** | 2.54 | **0.001** |  |  |  |  |  |  |  |  |  |  |  |  |  |  |  |  |
| **Dominant Model** | | | | | | | | | | | | |  | |  | |  | |  | | |  | | |  | |  |  |
| TA+AA | 1.13 | 0.647 | 1.14 | 0.596 | 1.28 | 0.344 | 1.03 | 0.916 | 1.20 | 0.477 | 1.43 | 0.168 |  |  |  |  |  |  |  |  |  |  |  |  |  |  |  |  |
| Age (years) | 0.99 | 0.364 | 1.00 | 0.991 | 0.99 | 0.377 | 1.00 | 0.956 | 1.01 | 0.242 | 1.01 | 0.223 |  |  |  |  |  |  |  |  |  |  |  |  |  |  |  |  |
| Urban | 4.46 | **<0.001** | 3.45 | **<0.001** | 3.69 | **<0.001** | 2.14 | **0.009** | 2.34 | **0.002** | 2.55 | **0.001** |  |  |  |  |  |  |  |  |  |  |  |  |  |  |  |  |
| **Recessive Model** | | | | | | | | | | | | |  | |  | |  | |  | | |  | | |  | |  |  |
| AA | 1.02 | 0.948 | 1.11 | 0.73 | 1.08 | 0.795 | 2.83 | **0.003** | 1.61 | 0.185 | 1.22 | 0.586 |  |  |  |  |  |  |  |  |  |  |  |  |  |  |  |  |
| Age (years) | 0.99 | 0.359 | 1.00 | 0.991 | 0.99 | 0.388 | 1.00 | 0.871 | 1.01 | 0.26 | 1.01 | 0.204 |  |  |  |  |  |  |  |  |  |  |  |  |  |  |  |  |
| Urban | 4.43 | **<0.001** | 3.41 | **<0.001** | 3.62 | **<0.001** | 2.03 | **0.016** | 2.25 | **0.002** | 2.46 | **0.001** |  |  |  |  |  |  |  |  |  |  |  |  |  |  |  |  |
| **FTO rs1421085** | | | | | | | | | | | | |  |  |  |  |  |  | |  |  | |  |  | |  |  |  |
| **Codominant Model** | | | | | | | | | | | | |  |  |  |  |  |  | |  |  | |  |  | |  |  |  |
| TC | 1.40 | 0.226 | 1.22 | 0.455 | 1.33 | 0.294 | 0.80 | 0.466 | 1.17 | 0.561 | 1.57 | 0.097 |  |  |  |  |  |  |  |  |  |  |  |  |  |  |  |  |
| CC | 1.08 | 0.824 | 1.47 | 0.237 | 1.79 | 0.093 | 2.99 | **0.005** | 2.02 | 0.070 | 1.81 | 0.133 |  |  |  |  |  |  |  |  |  |  |  |  |  |  |  |  |
| Age (years) | 0.99 | 0.442 | 1.00 | 0.973 | 0.99 | 0.41 | 1.00 | 0.900 | 1.01 | 0.285 | 1.01 | 0.244 |  |  |  |  |  |  |  |  |  |  |  |  |  |  |  |  |
| Urban | 4.61 | **<0.001** | 3.48 | **<0.001** | 3.73 | **<0.001** | 2.21 | **0.008** | 2.35 | **0.001** | 2.57 | **0.001** |  |  |  |  |  |  |  |  |  |  |  |  |  |  |  |  |
| **Dominant Model** | | | | | | | | | | | | |  | |  | |  | |  | | |  | | |  | |  |  |
| TC+CC | 1.28 | 0.327 | 1.30 | 0.294 | 1.46 | 0.138 | 1.03 | 0.916 | 1.20 | 0.477 | 1.43 | 0.168 |  |  |  |  |  |  |  |  |  |  |  |  |  |  |  |  |
| Age (years) | 0.99 | 0.411 | 1.00 | 0.941 | 0.99 | 0.439 | 1.00 | 0.956 | 1.01 | 0.242 | 1.01 | 0.223 |  |  |  |  |  |  |  |  |  |  |  |  |  |  |  |  |
| Urban | 4.53 | **<0.001** | 3.51 | **<0.001** | 3.78 | **<0.001** | 2.14 | **0.009** | 2.34 | **0.002** | 2.55 | **0.001** |  |  |  |  |  |  |  |  |  |  |  |  |  |  |  |  |
| **Recessive Model** | | | | | | | | | | | | |  | |  | |  | |  | | |  | | |  | |  |  |
| CC | 0.90 | 0.716 | 1.32 | 0.343 | 1.53 | 0.175 | 3.36 | **0.001** | 1.85 | 0.085 | 1.41 | 0.344 |  |  |  |  |  |  |  |  |  |  |  |  |  |  |  |  |
| Age (years) | 0.99 | 0.361 | 1.00 | 0.958 | 0.99 | 0.359 | 1.00 | 0.850 | 1.01 | 0.270 | 1.01 | 0.214 |  |  |  |  |  |  |  |  |  |  |  |  |  |  |  |  |
| Urban | 4.45 | **<0.001** | 3.42 | **<0.001** | 3.63 | **<0.001** | 2.22 | **0.008** | 2.33 | **0.002** | 2.49 | **0.001** |  |  |  |  |  |  |  |  |  |  |  |  |  |  |  |  |

Statistical analysis was done using logistic regression model, with adjustments for age and population (urban/rural). BMI: body mass index, WC: waist circumference, WHtR: waist to height ratio. High BMI: BMI ≥ 25, High WC: male’s wc ≥ 90 cm or female’s wc ≥ 80 cm, High WHtR: WHtR ≥ 0.5. The significant *p* values after Bonferroni correction are indicated in bold (*p* < 0.025).

Table S6. Models of FTO SNPs associations with obesity parameters in urban vs rural

|  | **Urban (n = 318)** | | | | | | **Rural (n = 294)** | | | | | |
| --- | --- | --- | --- | --- | --- | --- | --- | --- | --- | --- | --- | --- |
|  | **BMI** | | **WC** | | **WHtR** | | **BMI** | | **WC** | | **WHtR** | |
|  | *Estimates* | *p* | *Estimates* | *p* | *Estimates* | *p* | *Estimates* | *p* | *Estimates* | *p* | *Estimates* | *p* |
| **FTO rs9939609** | | | | | | | | | | | | |
| **Codominant Model** | | | | | | | | | | | | |
| TA | -0.44 | 0.451 | 0.41 | 0.754 | <0.01 | 0.946 | 1.04 | 0.071 | 2.32 | 0.092 | 0.02 | 0.085 |
| AA | 0.85 | 0.246 | 3.27 | 0.046 | 0.02 | 0.119 | 2.20 | **0.009** | 0.90 | 0.653 | 0.01 | 0.684 |
| Age (years) | 0.03 | 0.182 | 0.14 | **0.003** | <0.01 | **<0.001** | -0.04 | **0.010** | -0.09 | **0.022** | <0.01 | 0.484 |
| Male | 1.29 | **0.020** | 6.21 | **<0.001** | <0.01 | 0.976 | -0.07 | 0.897 | 2.90 | **0.019** | -0.02 | 0.039 |
| **Dominant Model** | | | | | | | | | | | | |
| TA+AA | -0.04 | 0.944 | 1.31 | 0.285 | 0.01 | 0.481 | 1.27 | **0.023** | 2.04 | 0.124 | 0.01 | 0.119 |
| Age (years) | 0.03 | 0.205 | 0.14 | **0.004** | <0.01 | **<0.001** | -0.04 | **0.012** | -0.09 | **0.020** | <0.01 | 0.463 |
| Male | 1.36 | **0.014** | 6.36 | **<0.001** | <0.01 | 0.939 | -0.04 | 0.939 | 2.86 | **0.021** | -0.02 | 0.036 |
| **Recessive Model** | | | | | | | | | | | | |
| AA | 1.09 | 0.097 | 3.04 | 0.038 | 0.02 | 0.088 | 1.53 | 0.043 | -0.59 | 0.743 | <0.01 | 0.694 |
| Age (years) | 0.03 | 0.192 | 0.14 | **0.003** | <0.01 | **<0.001** | -0.04 | **0.012** | -0.09 | 0.026 | <0.01 | 0.518 |
| Male | 1.28 | **0.021** | 6.22 | **<0.001** | <0.01 | 0.978 | -0.04 | 0.932 | 2.94 | **0.018** | -0.02 | 0.044 |
| **FTO rs1421085** | | | | | | | | | | | | |
| **Codominant Model** | | | | | | | | | | | | |
| TC | 0.05 | 0.937 | 1.48 | 0.255 | 0.01 | 0.372 | 1.09 | 0.058 | 2.51 | 0.069 | 0.02 | 0.053 |
| CC | 0.65 | 0.385 | 2.79 | 0.097 | 0.01 | 0.209 | 2.30 | **0.003** | 3.17 | 0.082 | 0.02 | 0.068 |
| Age (years) | 0.03 | 0.214 | 0.14 | **0.004** | <0.01 | **<0.001** | -0.04 | **0.011** | -0.09 | **0.023** | <0.01 | 0.496 |
| Male | 1.32 | **0.018** | 6.29 | **<0.001** | <0.01 | 0.976 | -0.07 | 0.885 | 2.83 | **0.022** | -0.02 | 0.033 |
| **Dominant Model** | | | | | | | | | | | | |
| TC+CC | 0.22 | 0.677 | 1.86 | 0.122 | 0.01 | 0.231 | 1.41 | **0.01** | 2.68 | 0.040 | 0.02 | 0.029 |
| Age (years) | 0.03 | 0.207 | 0.14 | **0.004** | <0.01 | **<0.001** | -0.04 | **0.016** | -0.09 | **0.024** | <0.01 | 0.513 |
| Male | 1.35 | **0.015** | 6.37 | **<0.001** | <0.01 | 0.938 | -0.04 | 0.934 | 2.84 | **0.021** | -0.02 | 0.034 |
| **Recessive Model** | | | | | | | | | | | | |
| CC | 0.63 | 0.361 | 2.03 | 0.189 | 0.01 | 0.326 | 1.65 | **0.016** | 1.68 | 0.304 | 0.01 | 0.286 |
| Age (years) | 0.03 | 0.213 | 0.14 | **0.004** | <0.01 | **<0.001** | -0.04 | **0.010** | -0.09 | **0.020** | <0.01 | 0.460 |
| Male | 1.32 | **0.018** | 6.29 | **<0.001** | <0.01 | 0.974 | -0.05 | 0.915 | 2.87 | **0.021** | -0.02 | 0.037 |

Statistical analysis was done using linear regression model, with adjustments for age and sex (male/female). BMI: body mass index, WC: waist circumference, WHtR: waist to height ratio. The significant *p* values after Bonferroni correction are indicated in bold (*p* < 0.025).

Table S7. Models of FTO SNPs associations with obesity categories in urban vs. rural

|  | **Urban (n = 318)** | | | | | | **Rural (n = 294)** | | | | | |
| --- | --- | --- | --- | --- | --- | --- | --- | --- | --- | --- | --- | --- |
|  | **High BMI** | | **High WC** | | **High WHtR** | | **High BMI** | | **High WC** | | **High WHtR** | |
|  | *Odds Ratio* | *p* | *Odds Ratio* | *p* | *Odds Ratio* | *p* | *Odds Ratio* | *p* | *Odds Ratio* | *p* | *Odds Ratio* | *p* |
| **FTO rs9939609** | | | | | | | | | | | | |
| **Codominant Model** | | | | | | | | | | | | |
| TA | 0.75 | 0.257 | 0.95 | 0.839 | 1.07 | 0.826 | 1.44 | 0.28 | 1.30 | 0.355 | 1.60 | 0.078 |
| AA | 1.16 | 0.647 | 1.66 | 0.127 | 1.75 | 0.151 | 2.68 | **0.024** | 1.09 | 0.832 | 1.14 | 0.732 |
| Age (years) | 1.01 | 0.554 | 1.03 | **0.007** | 1.04 | **0.001** | 0.99 | 0.148 | 0.99 | 0.283 | 0.98 | 0.036 |
| Male | 2.20 | **0.001** | 0.55 | **0.017** | 0.90 | 0.717 | 1.02 | 0.947 | 0.41 | **<0.001** | 0.71 | 0.154 |
| **Dominant Model** | | | | | | | | | | | | |
| TA+AA | 0.86 | 0.518 | 1.13 | 0.624 | 1.23 | 0.443 | 1.64 | 0.123 | 1.26 | 0.404 | 1.50 | 0.116 |
| Age (years) | 1.01 | 0.586 | 1.03 | **0.009** | 1.04 | **0.001** | 0.99 | 0.166 | 0.99 | 0.273 | 0.98 | 0.033 |
| Male | 2.24 | **0.001** | 0.57 | **0.023** | 0.92 | 0.78 | 1.04 | 0.891 | 0.41 | **<0.001** | 0.71 | 0.145 |
| **Recessive Model** | | | | | | | | | | | | |
| AA | 1.36 | 0.285 | 1.71 | 0.075 | 1.69 | 0.141 | 2.10 | 0.044 | 0.92 | 0.828 | 0.84 | 0.625 |
| Age (years) | 1.01 | 0.585 | 1.03 | **0.008** | 1.04 | **0.001** | 0.99 | 0.160 | 0.99 | 0.291 | 0.98 | 0.042 |
| Male | 2.17 | **0.001** | 0.55 | **0.017** | 0.91 | 0.726 | 1.02 | 0.936 | 0.41 | **<0.001** | 0.72 | 0.168 |
| **FTO rs1421085** | | | | | | | | | | | | |
| **Codominant Model** | | | | | | | | | | | | |
| TC | 0.93 | 0.768 | 1.11 | 0.693 | 1.13 | 0.660 | 1.50 | 0.233 | 1.31 | 0.337 | 1.82 | 0.026 |
| CC | 1.14 | 0.685 | 2.06 | 0.038 | 1.87 | 0.127 | 2.90 | **0.008** | 1.41 | 0.355 | 1.98 | 0.054 |
| Age (years) | 1.00 | 0.610 | 1.03 | **0.010** | 1.04 | **0.001** | 0.99 | 0.146 | 0.99 | 0.283 | 0.98 | 0.038 |
| Male | 2.19 | **0.001** | 0.54 | **0.015** | 0.90 | 0.721 | 1.01 | 0.966 | 0.40 | **<0.001** | 0.70 | 0.134 |
| **Dominant Model** | | | | | | | | | | | | |
| TC+CC | 0.99 | 0.954 | 1.32 | 0.245 | 1.29 | 0.337 | 1.81 | 0.064 | 1.34 | 0.280 | 1.86 | **0.015** |
| Age (years) | 1.01 | 0.599 | 1.03 | **0.009** | 1.04 | **0.001** | 0.99 | 0.190 | 0.99 | 0.289 | 0.98 | 0.039 |
| Male | 2.22 | **0.001** | 0.57 | **0.022** | 0.93 | 0.785 | 1.03 | 0.905 | 0.40 | **<0.001** | 0.70 | 0.136 |
| **Recessive Model** | | | | | | | | | | | | |
| CC | 1.19 | 0.567 | 1.95 | 0.037 | 1.75 | 0.143 | 2.25 | **0.016** | 1.20 | 0.585 | 1.38 | 0.304 |
| Age (years) | 1.00 | 0.610 | 1.03 | **0.010** | 1.04 | **0.001** | 0.99 | 0.140 | 0.99 | 0.269 | 0.98 | 0.034 |
| Male | 2.19 | **0.001** | 0.54 | **0.015** | 0.91 | 0.733 | 1.02 | 0.951 | 0.41 | **<0.001** | 0.71 | 0.148 |

Statistical analysis was done using logistic regression model, with adjustments for age and sex (male/female). BMI: body mass index, WC: waist circumference, WHtR: waist to height ratio. High BMI: BMI ≥ 25, High WC: male’s wc ≥ 90 cm or female’s wc ≥ 80 cm, High WHtR: WHtR ≥ 0.5. The significant *p* values after Bonferroni correction are indicated in bold (*p* < 0.025).

Table S8. Associations of FTO SNPs rs9939609/rs1421085 haplotypes with obesity

| **Total (N = 612)** | **BMI** | | **WC** | | **WHtR** | | **High BMI** | | **High WC** | | **High WHtR** | |
| --- | --- | --- | --- | --- | --- | --- | --- | --- | --- | --- | --- | --- |
|  | *Est.* | *p* | *Est.* | *p* | *Est.* | *p* | *Odds ratio* | *p* | *Odds ratio* | *p* | *Odds ratio* | *p* |
| Haplotype AC (38.2%) | 0.73 | **0.008** | 1.47 | **0.022** | 0.01 | 0.048 | 1.25 | 0.084 | 1.24 | 0.084 | 1.30 | 0.047 |
| Haplotype AT (3.4%) | -0.46 | 0.511 | -0.75 | 0.649 | -0.01 | 0.454 | 0.74 | 0.390 | 0.65 | 0.212 | 0.72 | 0.320 |
| Haplotype TC (2.4%) | -0.22 | 0.788 | 2.15 | 0.252 | 0.02 | 0.197 | 1.00 | 0.994 | 1.33 | 0.440 | 2.07 | 0.086 |
| Age (years) | -0.01 | 0.486 | 0.01 | 0.659 | <0.01 | **0.012** | 1.00 | 0.810 | 1.01 | 0.255 | 1.00 | 0.551 |
| Male | 0.83 | **0.026** | 5.21 | **<0.001** | <0.01 | 0.432 | 1.69 | **0.004** | 0.53 | **<0.001** | 0.92 | 0.656 |
| Urban | 2.84 | **<0.001** | 7.71 | **<0.001** | 0.04 | **<0.001** | 3.30 | **<0.001** | 2.93 | **<0.001** | 3.13 | **<0.001** |

Statistical analysis was done using linear regression model, while adjusting for age, gender (female/male) and population (urban/rural). The model used for linear regression: outcome ~ haplotype + age + gender + population. Haplotype AC: A allele in rs9939609 and C allele in rs1421085. Haplotype AT: A allele in rs9939609 and T allele in rs1421085. Haplotype TC: T allele in rs9939609 and C allele in rs1421085. BMI: body mass index, WC: waist circumference, WHtR: waist to height ratio. High BMI: BMI ≥ 25, High WC: male’s WC ≥ 90 cm or female’s WC ≥ 80 cm, High WHtR: WHtR ≥ 0.5. The significant *p*-values after Bonferroni correction are indicated in bold (*p* < 0.025).

Table S9. Associations of FTO SNPs haplotypes with obesity in male vs female.

|  | **Male (n = 326)** | | | | | | | | | | | |
| --- | --- | --- | --- | --- | --- | --- | --- | --- | --- | --- | --- | --- |
|  | **BMI** | | **WC** | | **WHtR** | | **High BMI** | | **High WC** | | **High WHtR** | |
|  | *Est.* | *p* | *Est.* | *p* | *Est.* | *p* | *Odds ratio* | *p* | *Odds ratio* | *p* | *Odds ratio* | *p* |
| Haplotype AC (39.9%) | 0.61 | 0.069 | 0.91 | 0.280 | <0.01 | 0.471 | 1.10 | 0.571 | 1.18 | 0.326 | 1.24 | 0.230 |
| Haplotype AT (3.9%) | -0.15 | 0.852 | -0.92 | 0.654 | -0.01 | 0.453 | 0.62 | 0.295 | 0.47 | 0.113 | 0.60 | 0.245 |
| Haplotype TC (2.7%) | -1.10 | 0.232 | 0.20 | 0.931 | <0.01 | 0.907 | 0.58 | 0.325 | 0.95 | 0.911 | 1.77 | 0.275 |
| Age (years) | -0.02 | 0.213 | -0.04 | 0.367 | <0.01 | 0.303 | 0.99 | 0.427 | 1.00 | 0.921 | 0.99 | 0.437 |
| Urban | 3.47 | **<0.001** | 9.44 | **<0.001** | 0.05 | **<0.001** | 4.52 | **<0.001** | 3.62 | **<0.001** | 3.90 | **<0.001** |
|  | **Female (n = 286)** | | | | | | | | | | | |
|  | **BMI** | | **WC** | | **WHtR** | | **High BMI** | | **High WC** | | **High WHtR** | |
|  | *Est.* | *p* | *Est.* | *p* | *Est.* | *p* | *Odds ratio* | *p* | *Odds ratio* | *p* | *Odds ratio* | *p* |
| Haplotype AC (36.1%) | 0.84 | 0.062 | 1.99 | 0.041 | 0.01 | 0.045 | 1.46 | 0.058 | 1.29 | 0.169 | 1.33 | 0.142 |
| Haplotype AT (2.7%) | -1.05 | 0.405 | -0.66 | 0.809 | -0.01 | 0.718 | 0.91 | 0.877 | 0.97 | 0.953 | 0.92 | 0.874 |
| Haplotype TC (2.0%) | 1.31 | 0.357 | 5.53 | 0.074 | 0.04 | 0.054 | 2.23 | 0.167 | 2.35 | 0.189 | 2.81 | 0.172 |
| Age (years) | -0.01 | 0.715 | 0.03 | 0.438 | 0.00 | 0.069 | 1.00 | 0.875 | 1.01 | 0.279 | 1.01 | 0.243 |
| Urban | 2.11 | **0.001** | 5.83 | **<0.001** | 0.02 | **0.007** | 2.22 | **0.007** | 2.38 | **0.001** | 2.58 | **0.001** |

Statistical analysis was done using linear regression model, while adjusting for age and population (urban/rural). The model used for linear regression: outcome ~ haplotype + age + population. Haplotype AC: A allele in rs9939609 and C allele in rs1421085. Haplotype AT: A allele in rs9939609 and T allele in rs1421085. Haplotype TC: T allele in rs9939609 and C allele in rs1421085. BMI: body mass index, WC: waist circumference, WHtR: waist to height ratio. High BMI: BMI ≥ 25, High WC: male’s WC ≥ 90 cm or female’s WC ≥ 80 cm, High WHtR: WHtR ≥ 0.5. The significant *p*-values after Bonferroni correction are indicated in bold (*p* < 0.025).

Table S10. Associations of FTO SNPs haplotypes with obesity in urban vs rural.

|  | **Urban (n = 318)** | | | | | | | | | | | |
| --- | --- | --- | --- | --- | --- | --- | --- | --- | --- | --- | --- | --- |
|  | **BMI** | | **WC** | | **WHtR** | | **High BMI** | | **High WC** | | **High WHtR** | |
|  | *Est.* | *p* | *Est.* | *p* | *Est.* | *p* | *Odds ratio* | *p* | *Odds ratio* | *p* | *Odds ratio* | *p* |
| Haplotype AC (37.5%) | 0.34 | 0.356 | 1.61 | 0.054 | 0.01 | 0.134 | 1.05 | 0.773 | 1.34 | 0.083 | 1.33 | 0.141 |
| Haplotype AT (4.3%) | -0.40 | 0.659 | -0.56 | 0.786 | -0.01 | 0.642 | 0.77 | 0.516 | 0.62 | 0.232 | 0.86 | 0.737 |
| Haplotype TC (1.6%) | -1.56 | 0.256 | -3.20 | 0.298 | -0.02 | 0.246 | 0.68 | 0.522 | 1.01 | 0.989 | 0.77 | 0.703 |
| Age (years) | 0.03 | 0.180 | 0.14 | **0.003** | <0.01 | **<0.001** | 1.01 | 0.563 | 1.03 | **0.008** | 1.04 | **0.001** |
| Male | 1.33 | **0.016** | 6.28 | **<0.001** | <0.01 | 0.976 | 2.22 | **0.001** | 0.56 | **0.021** | 0.91 | 0.733 |
|  | **Rural (n = 294)** | | | | | | | | | | | |
|  | **BMI** | | **WC** | | **WHtR** | | **High BMI** | | **High WC** | | **High WHtR** | |
|  | *Est.* | *p* | *Est.* | *p* | *Est.* | *p* | *Odds ratio* | *p* | *Odds ratio* | *p* | *Odds ratio* | *p* |
| Haplotype AC (38.9%) | 1.20 | **0.003** | 1.14 | 0.235 | 0.01 | 0.227 | 1.71 | **0.017** | 1.14 | 0.516 | 1.26 | 0.223 |
| Haplotype AT (2.3%) | -0.55 | 0.617 | -1.29 | 0.626 | -0.01 | 0.486 | 0.52 | 0.499 | 0.72 | 0.602 | 0.48 | 0.244 |
| Haplotype TC (3.1%) | 0.46 | 0.633 | 4.89 | 0.034 | 0.03 | 0.026 | 1.33 | 0.569 | 1.51 | 0.366 | 3.23 | 0.037 |
| Age (years) | -0.04 | **0.012** | -0.09 | **0.023** | 0.00 | 0.506 | 0.99 | 0.163 | 0.99 | 0.288 | 0.98 | 0.041 |
| Male | -0.05 | 0.920 | 2.80 | **0.023** | -0.02 | **0.032** | 1.02 | 0.937 | 0.40 | **<0.001** | 0.69 | 0.130 |

Statistical analysis was done using linear regression model, while adjusting for age and population (urban/rural). The model used for linear regression: outcome ~ haplotype + age + gender. Haplotype AC: A allele in rs9939609 and C allele in rs1421085. Haplotype AT: A allele in rs9939609 and T allele in rs1421085. Haplotype TC: T allele in rs9939609 and C allele in rs1421085. BMI: body mass index, WC: waist circumference, WHtR: waist to height ratio. High BMI: BMI ≥ 25, High WC: male’s WC ≥ 90 cm or female’s WC ≥ 80 cm, High WHtR: WHtR ≥ 0.5. The significant *p*-values after Bonferroni correction are indicated in bold (*p* < 0.025).
